# Two Cases of Suprachoroidal Hemorrhage after Implantation of an Ex-Press Miniature Glaucoma Device and an Intraocular Lens

**DOI:** 10.1155/2014/294921

**Published:** 2014-11-18

**Authors:** Evan B. Dreyer, Rebecca E. Dreyer

**Affiliations:** ^1^Glaucoma-Cataract Consultants Inc., 1145 Bower Hill Road, Pittsburgh, PA 15243, USA; ^2^Case Western Reserve University, Cleveland, OH 44106, USA

## Abstract

Suprachoroidal hemorrhage is a rare complication of ophthalmic surgery in general and of glaucoma filtration procedures in particular. We present herein two cases of suprachoroidal hemorrhage in aphakic patients after simultaneous implantation of an Ex-Press miniature glaucoma device and an intraocular lens. Although a rare complication, we have now seen two cases develop in previously aphakic patients when we have attempted to place an Ex-Press miniature glaucoma device in conjunction with placement of a secondary intraocular lens. These two cases suggest that greater caution is warranted when attempting to surgically implant an express mini shut in the aphakic patient.

Suprachoroidal hemorrhage is a rare complication of ophthalmic surgery in general and of glaucoma filtration procedures in particular. Systemic and ocular risk factors include increased age, bleeding disorders, elevated blood pressure, high preoperative intraocular pressure, myopia, pseudoexfoliation, and aphakia or pseudophakia [1–3]. We present herein two cases of suprachoroidal hemorrhage in aphakic patients after simultaneous implantation of an Ex-Press miniature glaucoma device and an intraocular lens [4, 5].

*Patient 1*. A 93-year-old Caucasian female has been followed in our practice since 1961. She underwent uncomplicated intracapsular cataract extractions with peripheral iridectomies in 1970, for both eyes. Her acuity was correctable to 20/20 in both eyes postoperatively. She developed open angle glaucoma, with an IOP of 26 OD and 25 OS and optic nerve changes in 1999. This was treated topically with IOPs ranging from 13 to 19 through 2007. In 2002, she developed systemic hypertension; this has been successfully controlled with hydrochlorothiazide only. In 2007, her acuity OD declined to 20/200 as a consequence of corneal edema, macular changes, and an increase in intraocular pressures to 34 mm Hg. Despite alterations in topical therapy and attempts at laser trabeculoplasty, her acuity declined to the hand motion range, and her IOP continued to climb to 50 in October, 2011. The decision was made to attempt placement of an Ex-Press P50 miniature glaucoma device. Because of her aphakic status, an anterior chamber intraocular lens was placed at the time of surgery in an effort to prevent the vitreous from clogging the shunt. Although consideration was given to performing a vitrectomy either before or simultaneously with the glaucoma procedures, little or no residual anterior vitreous was present. After consultation with a retina-vitreous surgeon, vitrectomy was deemed an unnecessary additional risk. The mini shunt was placed beneath a 2.5 mm × 3 mm rectangular trabeculectomy flap, following the procedure of Dahan and Carmichael [6]. A solution of 0.4 mg Mitomycin C was applied to the flap bed for one minute before placement of the device. The flap was secured tightly with two 10-0 nylon sutures at the two posterior corners of the flap. The anterior chamber was irrigated, and minimal fluid escaped below the flap. The conjunctiva was closed with interrupted 8-0 vicryl sutures. No viscoelastic was utilized during the procedure. Approximately twelve hours postoperatively, the patient developed severe pain in the operated eye. She presented for evaluation approximately six hours later. On the first postoperative day, the vision had declined to light perception. The anterior chamber was slightly shallow, and the intraocular pressure was 17 mm. The shunt and the lens were in good position. The wound was Seidel negative, and there was little if any evidence of a filtering bleb. It is impossible, of course, to determine the patient who has sustained a hypotonous episode between the time of surgery and this evaluation. Examination of the posterior pole was made with difficulty; dark choroidal effusions were present. The diagnosis of a suprachoroidal hemorrhage was made. The patient declined additional intervention until fourteen days had passed. At that time, the hemorrhage was drained through an inferotemporal scleral incision, approximately seven mm behind the limbus, and blood was removed from the suprachoroidal space. A second incision was made seven mm behind the limbus, superotemporally. The drainage was continued until no additional blood was expressible with mild pressure at the scleral incision, nor when the anterior chamber was re-inflated. Postoperatively, her vision improved to 20/200 where it has remained. Her intraocular pressure has remained in the low twenties with a limited topical regimen consisting of pilocarpine 4% applied QID, and timolol-dorzolamide applied BID.

*Patient 2*. A (now) forty-five-year-old Caucasian male underwent bilateral cataract extraction in 1967, when he was one year old. He has had a longstanding history of barely controlled IOP and nystagmus and he underwent bilateral cyclocryotheraphy in 2000. Vision was lost over time in the right eye as a consequence of glaucomatous damage; he was able to maintain acuity of 20/100 until 2010. At that point, he developed central retinal vein occlusion and neovascular glaucoma, with an IOP of 50. Repeat cyclocryoablation was performed with the injection of Avastin. Despite therapy with brimonidine, pilocarpine, timolol, dorzolamide, and acetazolamide, his IOP remained uncontrolled.

As described above (with Patient 1), an Ex-Press P50 miniature glaucoma device was implanted, except that Mitomycin C was applied for four minutes. A posterior chamber intraocular lens was sutured in place. As with the first patient, consideration was given to performing a vitrectomy either before or simultaneously with the glaucoma procedures. As above, after consultation with a retina vitreous surgeon, vitrectomy was deemed an unnecessary additional risk. The balance of the surgical procedure was as described above. That evening, the patient blew his nose and developed eye pain immediately. The following day, the patient's vision was light perception. The anterior chamber was slightly shallow, and the lens and mini shunt were in good position. The wound was Seidel negative; no filtering bleb could be appreciated. Corneal edema was present. The intraocular pressure was 23 mm. As with the first patient, a perioperative hypotonous episode cannot be ruled out. A B-scan ultrasound was obtained. The scan showed an effusion in the suprachoroidal space with a density consistent with blood. At least two quadrants appeared to be involved. The diagnosis of a suprachoroidal hemorrhage was made, and the suprachoroidal space was drained of blood. Unfortunately, visual acuity has remained light perception only.

The precise etiology underlying a suprachoroidal hemorrhage remains unknown; various risk factors have been identified. Although a rare complication, we have now seen two cases develop in aphakic patients when we have attempted to place an Ex-Press miniature glaucoma device in conjunction with placement of a secondary intraocular lens. One cannot ignore the other risk factors that each patient evidenced (as described above) but we have not seen any other suprachoroidal hemorrhages in conjunction with repeated use of the express mini shunt, in dozens of patients with similar risk factors. What changes in fluid and hemodynamics are occasioned by the simultaneous placement of an intraocular lens remains a mystery. It is of course possible that it is the aphakic status alone that underlies the greater risk of suprachoroidal hemorrhage.

However, these two cases suggest that greater caution is warranted when attempting to surgically implant an express mini shut in the aphakic patient.
